# Gamma-Range Auditory Steady-State Responses and Cognitive Performance: A Systematic Review

**DOI:** 10.3390/brainsci11020217

**Published:** 2021-02-10

**Authors:** Vykinta Parciauskaite, Jovana Bjekic, Inga Griskova-Bulanova

**Affiliations:** 1 Life Sciences Centre, Institute of Biosciences, Vilnius University, Sauletekio ave 7, LT-10257 Vilnius, Lithuania; vykinta.parciauskaite@stud.gmc.vu.lt; 2 Human Neuroscience Group, Institute for Medical Research, University of Belgrade, Dr Subotića 4, 11000 Belgrade, Serbia; jovana.bjekic@imi.bg.ac.rs

**Keywords:** auditory steady-state response, ASSR, 40 Hz, gamma range, cognitive functions, working memory, attention, cognitive flexibility

## Abstract

The auditory steady-state response (ASSR) is a result of entrainment of the brain’s oscillatory activity to the frequency and phase of temporally modulated stimuli. Gamma-range ASSRs are utilized to observe the dysfunctions of brain-synchronization abilities in neuropsychiatric and developmental disorders with cognitive symptoms. However, the link between gamma-range ASSRs and cognitive functioning is not clear. We systematically reviewed existing findings on the associations between gamma-range ASSRs and cognitive functions in patients with neuropsychiatric or developmental disorders and healthy subjects. The literature search yielded 1597 articles. After excluding duplicates and assessing eligibility, 22 articles were included. In healthy participants, the gamma-range ASSR was related to cognitive flexibility and reasoning as measured by complex tasks and behavioral indicators of processing speed. In patients with schizophrenia, the studies that reported correlations found a higher ASSR to be accompanied by better performance on short-term memory tasks, long-term/semantic memory, and simple speeded tasks. The main findings indicate that individual differences in the gamma-range ASSR reflect the level of attentional control and the ability to temporary store and manipulate the information, which are necessary for a wide range of complex cognitive activities, including language, in both healthy and impaired populations.

## 1. Introduction

Understanding the role of temporal brain synchronization in cognitive functioning has been a subject of intense research for many years [1,2]. Brain electrophysiological measures, such as electroencephalography (EEG) and magnetoencephalography (MEG), provide cost-effective, non-invasive techniques to explore the occurrence of neural synchronization and temporal states [3,4]. In this perspective, attention has been drawn to gamma-range (30–80 Hz) activity due to its association with cognitive performance [5,6]. A large range of cognitive functions, such as information-processing speed, working memory, abstract reasoning, and verbal abilities [7], which are related to gamma activity [8,9], are impaired in patients with neuropsychiatric and developmental disorders like schizophrenia [10], Alzheimer’s disease [11,12], dyslexia [13], aging [14], etc. Along with the observed dysfunctions in cognitive performance, the impaired gamma oscillations were also frequently reported in different patient groups [14,15].

One of the EEG/MEG techniques used to explore individual differences in neural synchronization is the method of auditory steady-state response (ASSR) [16]. The ASSR is an electrophysiological response of the brain that synchronizes to the frequency and phase of rapid, periodic auditory stimuli delivered in trains of clicks [17], broad-band noise [18], amplitude-modulated (AM) tones [19], or sounds modulated with chirps [20,21]. The response to auditory stimulation reaches the greatest magnitude with the presentation rates within the gamma range, especially around 40-Hz [22,23]. The sources of ASSR are located in the medial part of the primary auditory cortex [24,25,26] with a contribution from thalamic or corticothalamic sources [27,28]. However, more recent human [29,30] and animal studies [31,32] show a significant involvement of prefrontal cortex in gamma-range ASSR generation.

Hence, the ASSR is an effective tool to evaluate the state of synchronous oscillations and temporal information transfer in neural networks [33,34], with 40-Hz ASSRs being commonly used to evaluate abilities to generate gamma-range activity. The 40-Hz ASSR has been proposed as a biomarker of schizophrenia [16,35], as the impairments of 40-Hz ASSRs have been consistently reported across the schizo-bipolar spectrum [36,37,38]. Moreover, 40-Hz ASSRs are viewed as indexing neurochemical excitation/inhibition balance in the brain [39] maintained by *N*-methyl-d-aspartate (NMDA) and γ-aminobutyric acid (GABA) systems, as shown by animal studies [40,41,42].

Despite its relevance to auditory processing, it is often argued that the 40-Hz ASSR indexes impairments of some cognitive domains, as observed in neuropsychiatric patients [43,44]. However, there is no firm conclusion on the functional level regarding what ASSRs test. In the literature, there are two prevailing interpretations of gamma-range ASSRs: (1) ASSRs are viewed as indexing merely sensory processes and reflecting the integrity of auditory circuits [45,46]; and (2) ASSRs are indexing globally synchronized neural activity and information transfer [43,44]. Therefore, the functional role of gamma-range ASSRs and the way they can translate into individual differences in cognitive functioning is still unknown. Since gamma-range ASSRs measure physiological functioning of the auditory system, it is reasonable to expect that they are also related to cognitive/perceptual abilities in the auditory and speech domains. However, if gamma-range ASSRs index more general processes, then they may be associated with more general measures of cognition (e.g., non-verbal abilities, working memory, executive functioning, processing speed, etc.). Finally, gamma-range ASSRs may index both audition-related functioning and more general cognitive processes.

Nonetheless, a limited number of previous studies have directly addressed the relationship between gamma-range ASSRs and cognitive functions, even though the association can be expected based on the common underlying mechanisms. For example, 40-Hz ASSRs are sensitive to the levels of arousal [47,48] and attention [49,50,51], with both playing an important role in higher-level cognitive functioning [52]. A widely distributed network of sources, located in the cortical and subcortical regions, is active in response to 40-Hz stimulation [27,53,54]. Finally, ASSRs were shown to correlate with the level of cognitive impairment in clinical populations [55,56]. Nevertheless, the results of correlational analyses relating gamma-range ASSRs and cognitive functions are inconsistent, and factors contributing to the discrepancies have not been established yet. Therefore, to gain a better understanding and foster ASSR usage as an individual biological marker for disorders with prevailed cognitive symptoms, the relationship between gamma-range ASSRs and cognitive abilities needs to be investigated further. Hence, to outline the current state of knowledge, we aimed to systematize and critically evaluate previous studies addressing the relationships between gamma-range ASSRs and cognitive functions.

## 2. Methods

This systematic review was performed in accordance with the Primary Reporting Items for Systematic Reviews and Meta-Analyses (PRISMA) Statement [57].

### 2.1. Literature Search

**Figure 1:**
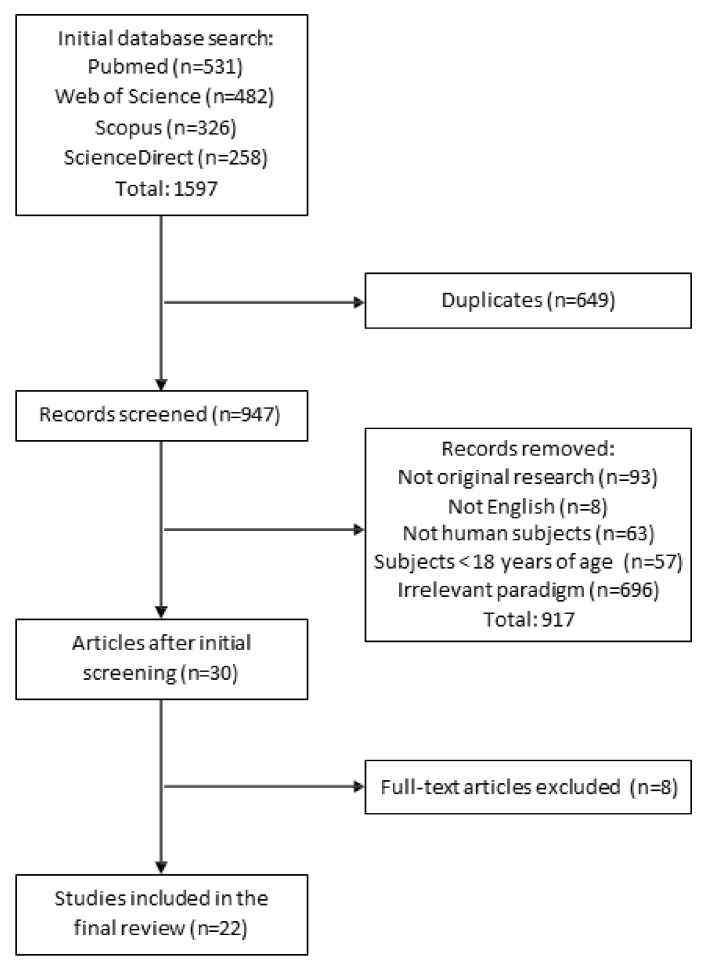
The schema of the search process and study selection.

Literature was collected using online searches in the PubMed, Web of Science and Scopus databases. The search was performed from June 2020–January 2021 and the keywords included “auditory amplitude modulated response”, “auditory steady state response”, “auditory entrainment”, “cognitive task”, “behavioral task”, “psychological task”, “verbal task”, “attention”, “cognition”, and “memory”. A manual search among the reference lists of included papers was also conducted to identify potentially relevant reports. All titles and abstracts were scanned for selection criteria. When the abstract provided insufficient information, the methods section of the article was reviewed. The selection-procedure flowchart is presented in Figure 1. The included studies were checked by the first and the last authors. When a disagreement arose, the second author’s opinion was sought.

### 2.2. Study Selection

The following inclusion criteria for study selection were used: (1) the participants were adults ≥ 18 years old; (2) EEG/MEG methods with gamma-range (30–80 Hz) auditory stimulation were used; (3) a behavioral assessment of cognitive performance was performed (4); a statistical association between ASSR measures and cognitive performance was reported; and (5) the article reported original research. Since this is the first systematic review of the cognitive correlates of gamma-range ASSRs, to be as inclusive as possible, studies in various neuropsychiatric and developmental disorders (e.g., schizophrenia, Alzheimer’s disease, dyslexia, etc.) were included. Original articles that were not found in the specified search but were cited or recommended by selected studies, or were known by the review authors and met the inclusion criteria, were included as well. The following papers were excluded: (1) animal studies; (2) studies measuring ASSRs in frequencies other than gamma-range; (3) studies not using adequate cognitive evaluation methods, i.e., not using cognitive tests/tasks or neuropsychological assessment tools; (4) studies in which ASSRs were collected during altered states (e.g., during high-cognitive-demand tasks, sleep, anesthesia, or hallucinations); (5) studies in which ASSRs could be affected by brain-stimulation techniques (e.g., tACS, TMS); (6) papers published in non-English languages. When papers were not accessible as a full-text version or lacked necessary information, efforts were made to retrieve the missing data by contacting the authors.

### 2.3. Data Extraction

For each study, the following information was extracted (Table 1): (1) sample (type, size, age, and gender composition); (2) neurocognitive assessment method (i.e., tasks that were used to assess cognitive performance); (3) auditory stimulation settings (stimulation frequencies, type, number of repetitions, duration); (4) the EEG/MEG assessment (measure, site, latency); and (5) the correlation between measure(s) of ASSRs and neurophysiological measure(s). To systematize the results, we grouped the neurocognitive performance assessment tasks that were used in the included studies into higher-order cognitive domains (Table 2): composite measures of global cognitive functioning, attentional control and executive functions, short-term and working memory, cognitive flexibility and reasoning, language abilities, and motor abilities. It is important to note that this classification is by no means an exhaustive list of well-established and validated assessment tools for listed cognitive functions, but only an attempt to systematize the methods used in included studies and overcome the disparity in the assessment tools that were used.

### 2.4. Quality Evaluation

The quality of included articles was rated in line with guidelines by the Cochrane handbook for systematic reviews of interventions [77] by first and last authors. When a disagreement arose, the second author’s opinion was sought. This assessment is primarily focused on the reporting bias with respect to key aspects of the study from the perspective of its reproducibility and replicability. The scale was adapted to capture major sources of bias, and each study was rated depending on the amount and quality of information that was provided in the article (see Supplementary Material).

## 3. Results

The literature search yielded 1597 articles. After excluding duplicates and studies that did not meet the inclusion criteria, 22 articles were included in this systematic review (Figure 1). Twelve out of 22 studies defined the ASSR association to the cognitive correlates as one of the primary study purposes; the remaining 10 reports presented ASSR–cognitive correlates as a non-primary topic.

### 3.1. Methodological Characteristics and Assessment of Selected Studies

Most of the studies examined multiple cognitive domains and used neuropsychological batteries: BACS [44,59,69,70], WAIS [75], ADAS-cog subscale [55], MATRICS MCCB [76], or different subtests from various batteries like WAIS [73,74] and PEBL [71]. Three of the studies evaluated only global cognitive functioning [59,61,69]. In contrast, some studies targeted at specific functions, e.g., short-term and working memory, applying digit span [72] or phonological awareness measured by a Spoonerism task [62].

The EEG/MEG response to repetitive click stimulation in the gamma frequency range was assessed in the majority of included studies; however, variations in stimulation duration, stimulus features, and inter-stimulus interval settings, as well as acquisition methods, were detected (Table 1). Several studies used amplitude-modulated sounds [60,62,75], or chirp-based stimulation [58,68]. The main ASSR outcome measures were power and phase synchronization/consistency; two studies provided signal-to-noise ratio evaluations [60,62], and one study provided individual gamma peak frequencies [58]. Most studies focused on the evaluation of the response during the entire stimulation duration [43,55,59,62,63,64,65,66,67,69,72,75]. Rass et al., Sun et al., and Gaskins et al. did not include the early response (0–100 ms) part [60,73,74,76]. Murphy et al., Tada et al., and Parčiauskaite et al. evaluated only the late-latency gamma (starting at 200 ms after stimulus onset) activity [44,70,71]. Finally, Arrondo et al. and Lehongre et al. focused on the time window of the maximal gamma response occurrence [58,68]. Three of the studies utilized MEG recordings [68,70,75]. EEG recordings in the selected reports were either performed with the nose serving as a reference, or electrodes were average referenced [44,63,65,66,67,72,76]. The EEG results were reported mostly for fronto-central locations, with the exception of van Deursen et al. and Hirtum et al., who analyzed temporal and temporoparietal locations [55,62].

Overall, the majority of the included studies were characterized by a low risk of reporting bias, with the exception of Bartolomeo et al. [59] and Gaskins et al. [60], who did not report paradigm settings in a sufficient manner. 

### 3.2. Correlations between ASSR and Cognitive Performance

The last column in Table 1 shows the correlation outcomes between performance on cognitive tasks and ASSR measures as reported in the included studies. The studies yielded the full range of effect sizes, with correlations from 0.13 to 0.76 (median correlation: 0.43). Still, it should be noted that nonsignificant correlations have seldom been reported in sufficient detail. To increase transparency and allow for future meta-analytical studies, it is advisable that the authors fully report on statistics for nonsignificant effects. The power of most studies was only sufficient to detect correlations higher than 0.50, with the exception of Kirihara et al. [67] and Koshiyama et al. [65,66,67], who performed their analyses on large samples, and thus were able to detect even correlations in the range of 0.10–0.20. Therefore, it is difficult to estimate the real strength of the relationship between ASSR and different cognitive domains.

In healthy participants, the gamma-range ASSR was related to cognitive flexibility and reasoning as measured by complex tasks such as Tower of London [71], Similarities [73], and the Mazes Test [76]. Additionally, the ASSR was related to behavioral indicators of processing speed, i.e., performance on the Trial making test [76] and Symbol coding [74].

Five out of 16 studies that assessed patients with psychotic symptomatology (schizophrenia, schizoaffective disorder, schizotypal personality disorder) showed no relationship between gamma-range ASSR and cognitive performance [59,61,64,69,70]. The studies that reported correlations found a higher ASSR to be accompanied by better performance on short-term memory tasks (such as Digit span and Letter–Number sequencing) [43,65,66,67,72], tasks tapping speeded access to long term/semantic memory (like Verbal fluency) [63] or simple speeded tasks (like Symbol coding) [44]. However, observations of complex reasoning tasks such as the Mazes test [76], Similarities (form WAIS-III battery) [74], and Tower of London [44] were not very consistent.

In other patient groups, gamma-range ASSRs were indicative of impairment in disease-relevant cognitive domains. Namely, studies that assessed language abilities in dyslexia reported a negative correlation with phonological awareness (i.e., performance on a Spoonerism task) and phonological fluency (as measured by RAN), as well as literacy and nonword repetition [62,68]. Furthermore, better overall functioning assessed with ADAS-cog was related to higher 40-Hz ASSRs in patients with mild Alzheimer’s disease [55]. Patients with multiple sclerosis who performed better on different cognitive tasks from BRB-N tended to respond at higher gamma frequencies [58]. Still, it should be noted that a study assessing bipolar patients found no relationship between ASSR and cognitive performance measured by several WAIS-III subtests [73].

## 4. Discussion

Impaired cognitive performance is frequently reported in patients with neuropsychiatric disorders, and is accompanied by aberrant gamma activity [78,79]. As a method of exploring individual differences in the ability to generate and sustain gamma-range activity, EEG/MEG-based auditory steady-state responses (ASSRs) are used [16]. However, the functional relationship between gamma-range ASSRs and cognitive functioning, and their link to individual differences in performance/abilities, is highly unresolved. This review aimed to evaluate the current state of knowledge on the associations between gamma-range ASSRs and cognitive functions as measured by various cognitive tests or batteries. The literature search was carried out in order to collect, systematize, and critically evaluate previous studies that assessed ASSRs within the 30–80 Hz range and various cognitive domains in the same study sample.

Twenty-two articles were included and analyzed in this review. An absolute majority of the studies were performed with an aim of ASSR evaluation in clinical populations and used wide array of tasks to tap into different cognitive functions. For comparison purposes within this review, the cognitive tasks used in the included studies were grouped into higher-order cognitive domains. It is important to point out that most of the studies used several tasks, but measured specific, and often narrow, cognitive domains. Apart from Rojas et al. [75], who used an abbreviated WAIS, none of the studies conducted a comprehensive cognitive assessment using either a full WAIS or set of test/tasks that would cover all aspects of cognitive abilities as they are defined by well-established accepted models, e.g., the Cattell–Horn–Carroll theory of cognitive abilities [80,81,82]. However, a comprehensive assessment of cognitive functioning was performed for clinical populations using condition-specific batteries in Sun et al., Murphy et al., Leonhardt et al., and Bartolomeo et al. [59,69,70,76]. Thus, the quality evidence of the relationship between cognition and ASSRs is currently higher for pathological than for normal functioning. To that point, only a few studies with large enough samples to reliably detect even smaller effect sizes found effects in clinical subjects, but not a healthy control group [64,65,66,67]. Smaller sample sizes tended to artificially inflate correlations, while the fact that most studies related very specific EEG measures to very general cognitive measures theoretically resulted in lower effect sizes. Therefore, based on the current evidence, it is difficult to reliably estimate the strength (or even existence) of the relationship between ASSRs and different cognitive functions.

The most frequent associations of gamma-range ASSRs were identified in the outcomes for the tasks assessing processing speed and short-term/working memory tasks, covering both efficiency and timing aspects of the performance. For example, the Symbol coding task was used in six studies [27,47,48,49,54,55]; in three of those, performance of the task was positively associated with measures of ASSR in patients with schizophrenia [44,74], multiple sclerosis [58], and healthy controls [74]. Similarly, the Digit span test was employed in seven studies [44,63,68,72,73,74,76]. However, it was positively associated to gamma-range ASSR measurements in two studies of schizophrenia groups [72,74] and first-degree relatives of patients [72], and negatively in one study of dyslexia [68]. The Letter–Number sequencing task was employed in five reports [43,64,65,66,67], four of which showed a positive association between task performance in patients with schizophrenia and 40-Hz ASSR measures. These results might imply that individual differences in gamma-range ASSRs reflect the individual differences in the ability to focus attention, and maintain and manipulate the information in short-term memory storage. However, it appears that the associations are evident mostly in patient groups, where short-term/working memory is affected.

Additionally, performance on several tasks tapping cognitive flexibility and reasoning correlated with measures of gamma-range ASSRs [71,74,76] in healthy controls [71,74,76] and patients with schizophrenia [74,76]. However, tasks evaluating cognitive flexibility and reasoning are defined by high versatility, and the functions they assess are intricately covering attentional control/executive functioning and memory processes [83,84]. This makes evaluation of a particular aspect that is contributing to the observed relationship difficult. For example, the moves on the Tower of London task (ToL), as assessed in reviewed papers, were positively correlated to the synchronization levels of gamma-range activity [71]. Cazalis et al. proposed that standard performers on ToL might need to put the higher load on working memory to perform the task, compared to superior performers [85]. Should that be the case, a positive relationship between gamma-range ASSRs and moves in ToL could highlight the working-memory-related aspects of the task. This assumption is indirectly supported by the speeded performance on short-term memory tasks (such as Digit span and Letter–Number sequencing) [43,65,72] and long term/semantic memory tasks (like Verbal fluency) [63], as observed in patients with psychotic symptomology, who display stronger/more precise gamma-range ASSRs.

Furthermore, studies designed to assess the relationship between gamma-range ASSRs and the degree of language impairment in dyslexia reported significant associations with several tests, thus suggesting stronger impairment of ASSRs with debilitated language function [62,68]. These results are consistent with reported links between ASSRs and behavioral outcomes of speech recognition [86,87]. Correlation between ASSR and language impairment may be attributed to the altered temporal sampling [33], but might also be a reflection of the common core functions, such as attentional control and the ability to maintain and manipulate content in short-term memory storage. Namely, language comprehension and production strongly depend on the temporary storage and processing of information, i.e., working memory [88]. This is especially prominent in different language disorders [89]. Therefore, it is plausible that the observed relationship between gamma-range ASSRs and language performance does not reflect differences in linguistic abilities per se, but rather stems from individual differences in a more fundamental ability to process information. To untangle this issue, future studies focusing on language performance should include a behavioral assessment of working or short-term memory to examine if the relationship between gamma-range ASSRs and language performance is function-specific or stands as a reflection of a more general ability. In addition, further research is needed to explore the relationship between language and ASSRs in different populations; the association might be of importance in different neuropsychiatric conditions, since the positive correlation between 40-Hz ASSRs and verbal fluency was observed by Kim et al. in schizophrenia patients as well [63].

Importantly, several studies not included in our review reported the relationship between auditory cognition assessed with a gap-detection task, and the preferred frequency of gamma in response to periodic stimulation [21,90]. Studies evaluated the resonant frequency of gamma activity that was also the focal point of the work included in the review [58]. The estimation of preferred gamma frequency is possible using both classical single-frequency stimulation [91] and as a response to the specific type of periodic stimulation with stimulation frequencies spanning a wide range [21,58]. This approach might be particularly promising in clinical testing [20,92], as it allows fast estimation of the individual properties of networks involved in response that might display associations to cognitive functioning [58]. The individual resonant frequencies within the gamma range were negatively related to the speed on attentional control and executive tasks, as shown in patients with multiple sclerosis [58]. This observation is in agreement with a positive relationship between peak gamma frequencies and working-memory performance over the stages of sedation with anesthesia [93,94]. Altogether, the results advise that the state of neural networks defining individual gamma frequencies may relate to the temporal resolution, and to the individual parameters of information-processing speed and performance efficiency.

Even though it seems that differences in gamma-range ASSRs reflect a central aspect of cognitive functioning, i.e., attentional control and information manipulation in both healthy and clinical populations, the available evidence is far from conclusive. It is important to highlight that the reviewed studies utilized various cognitive tasks in which gamma activity is expected to be involved. However, it was not the direct aim of the studies to explain how auditory cortical responses relate to each of those tasks and to shed the light on cognitive processes involved in those tasks. This may be one of the reasons for the lack of systematic assessment of the cognitive performance in relation to ASSRs. Furthermore, several methodological aspects could have contributed to the discrepancy of obtained results. Though the stimulation parameters for ASSRs were relatively consistent when responses to 40 Hz were assessed, the cognitive testing protocols were substantially diverse in the available body of literature. However, even with the same stimulation settings, and ASSR and cognitive-assessment approaches, different correlational outcomes were reported [43,64,65,73,74]. This suggests that inter-individual subject’s characteristics, such as age or gender, might have a moderation-like effect, as these are known to affect both cognitive performance [95,96] and ASSRs [64,97,98]. Importantly, differences in the momentarily state of arousal/attention levels [99,100], prevailing psychopathology [46], medication regimen [101], the general level of global functioning, and the stage of disease in neuropsychiatric patients [56,102] could have affected the relationships. Also, the gamma-range ASSR measures could potentially be compromised by myogenic and micro-saccadic activity [103] and may influence some of the results between ASSRs and cognitive processes reviewed in the manuscript. Therefore, future studies should adopt the designs that enable exploring the magnitude of the moderation effects of these variables.

## 5. Conclusions

Gamma-range ASSRs were associated with the outcomes of short-term and working-memory tasks, covering both efficiency and timing aspects of the performance, and with the outcomes on tasks aimed at evaluating processing speed. Additionally, performance on several tasks to tap cognitive flexibility and reasoning correlated with measurements of gamma-range ASSRs, indirectly supporting an association with attentional control/executive functioning and memory. Finally, a possible link of ASSRs with results of language-tapping tests was observed in dyslexia. We propose that individual differences in gamma-range ASSRs reflect the level of attentional control and the ability to temporarily store and manipulate the information, which is necessary for a wide range of complex cognitive activities in different clinical populations.

This review points out several important directions for future research. First, the assessment of behavioral effects alongside physiological measures, though indirectly, may provide important insights into the underlying mechanisms behind brain functioning and lead to more informative interpretation of results. Second, the heterogeneity of the methods for cognitive assessment and lack of systematic selection of behavioral tasks make it difficult to directly compare and evaluate effects presented in different studies. Therefore, future research would benefit form more theory- and model-driven selections of the cognitive tasks. Moreover, inclusion of several tasks tapping into different cognitive functions would enable a dissociation between different cognitive functions in relation to ASSRs. Third, exploring the same relationships in different patient groups may uncover the regularity between cognitive and physiological deficits that are common across different disorders. Finally, we would like to encourage studies specifically designed to test the hypothesis that gamma-range ASSRs reflect individual differences in working-memory ability and performance.

## Figures and Tables

**Table 1:** Characteristics of the studies included in the review.

	Article	Sample Size; Males/Females; Mean Age or Age Range in Years (Standard Deviation)	Neuropsychological Tasks Used	Stimuli Frequencies; Type; Train Duration; Number of Stimuli	ASSR Measures and Site	Correlations
**1**	Arrondo et al. 2009 [58]	Healthy controls: 22; n/a (similar) Patients with multiple sclerosis: 27; 10/17; 44.11 (11.45)	Brief repeatable battery-neuropsychological (BRB-N): Bushke selective reminding test (SRT); 10/36 Spatial recall test (SPART); Oral version of the symbol digit modalities test (SDMT); Paced auditory serial addition task with a 3 s interval (PASAT-3); Semantic word list generation (WLG)	1–120 Hz; chirp; 1.61 s; 500 sweeps	EEG Frequency and amplitude of the maximal response; at Fz and Cz	Healthy controls: n.s. Patients with multiple sclerosis: SDMT and the frequency of the maximal amplitude-following responses around 40-Hz (*r* = 0.524, *p* = 0.010); PASAT-3 and the frequency of the maximal amplitude-following responses around 40-Hz (*r* = 0.483, *p* = 0.012); WLG and the frequency of the maximal amplitude-following responses around 40-Hz (*r* = 0.437, *p* = 0.023)
**2**	Bartolomeo et al. 2019 [59]	Healthy controls: 16; 14/5; 22.9 (3.6) Early phase of psychosis: 34; 24/7; 22.0 (4.3)	The Brief Assessment of Cognition in Schizophrenia (BACS)	40 Hz; clicks; 500 ms; 8 trials	EEG Power; at Fz	Healthy controls: n.s. Early phase of psychosis: n.s.
**3**	Gaskins et al. 2019 [60]	Healthy younger subjects: 15; n/a; 22.3 (2.7) Healthy older subjects: 15; n/a; 70.3 (3.8)	Digit Symbol Coding and Digit Symbol Search	40, 80 Hz; AM tone; 300 ms; 1024 sweeps	EEG SNR; at Cz	Healthy younger subjects: n.s. for 80-Hz ASSR; n/a for 40-Hz ASSR Healthy older subjects: n.s. for 80-Hz ASSR; n/a for 40-Hz ASSR
**4**	Hirano et al. 2020 [61]	Healthy controls: 24; 20/4; 44.1 (7.3) Chronic stage schizophrenia: 23; 19/4; 45.6 (9.1)	Information subscale of the Wechsler Adult Intelligence Scale–Fourth Edition (WAIS-IV)	20, 30, and 40 Hz; clicks; 500 ms	EEG PLF, evoked power, induced power; tangential and radial dipoles in each hemisphere above primary auditory cortex	Healthy control: n.s. Chronic stage schizophrenia: n.s.
**5**	Hirtum et al. 2019 [62]	Healthy controls: 18; 8/10; 18–25 years Dyslexia: 20; 10/10; 18–25 years	Literacy; Spoonerisms task; Random Automatized Naming (RAN)	40 Hz; AM tone; 300 ms; 300 epochs	EEG SNR; temporal-parietal and occipital regions: left (TP7, P1, P3, P5, P7, P9, PO3, PO7, O1) and right (TP8, P2, P4, P6, P8, P10, PO4, PO8, O2)	Healthy controls: n.s. Dyslexia: Literacy and 40-Hz neural background activity in the right hemisphere (*r* = −0.35, *p* = 0.033); Spoonerisms task and 40-Hz neural background activity in the right hemisphere (*r* = −0.39, *p* = 0.017); RAN and 40-Hz neural background activity in the right hemisphere (*r* = −0.39, *p* = 0.017)
**6**	Kim et al. 2019 [63]	Healthy controls: 30; 13/17; 43.33 (12.95) Schizophrenia: 33; 16/17; 42.21 (10.99)	Trail Making Test-A and B; verbal fluency test; Korean-Auditory Verbal Learning Test (K-AVLT)	40 Hz; clicks; 500 ms; 150 trials	EEG Mean evoked power, ITC; at Cz	Healthy controls: n.s. Schizophrenia: Verbal fluency mean evoked power at 40-Hz (*r* = 0.223, *p* = 0.019)
**7**	Kirihara et al. 2012 [64]	Healthy controls: 188; 94/94; 43.9 (11.1) Schizophrenia: 234;182/52; 44.5 (8.8)	The Wide Range Achievement Test 3 Reading subtest; California Verbal Learning Test (CVLT-2 list A, 1–5 total score and the delayed free recall indices); Wisconsin Card Sorting Test (WCST-64); Letter-Number Sequencing (LNS) test forward and reordering conditions	30, 40 Hz; clicks; 500 ms; 200 trials	EEG Amplitude, ITC, cross frequency coupling, modulation index; at FCz	Healthy controls: n.s. Schizophrenia: n.s.
**8**	Koshiyama et al. 2020a [65]	Healthy controls: 283; 139/144; 44.5 (11.4) Schizophrenia: 428; 309/119; 44.5 (9.5)	Letter-Number Sequencing (LNS), California verbal learning test (CVLT-2 list A 1–5 total score), Wisconsin Card Sorting Test (WCST, perseverative responses)	40 Hz; clicks; 500 ms; 200 trains	EEG ERSP at Fz	Healthy control: n.s. Schizophrenia: 40-Hz ASSR predicted LNS scores (standardized coefficient *β* = 0.17, *p* < 5.5 × 10^−4^) and CVLT scores (*β* = 0.16, *p* < 1.1 × 10^−3^)
**9**	Koshiyama et al. 2020b [66]	Healthy controls: 293; 141/152; 44.7 (11.4) Schizophrenia: 427; 309/118; 45.5 (9.5)	Letter-Number Sequencing (LNS), Letter-Number Span (LN Span), California verbal learning test (CVLT-2 list A 1–5 total score), Reading subtest of the Wide Range Achievement Test-3 (WRAT)	40 Hz; clicks; 500 ms; 200 trains	EEG ITC, 8 dipoles above primary auditory cortex	Healthy control: n/a Schizophrenia: LN Span scores with ITC of 40-Hz ASSR in the right temporal cortex (*r* = 0.16, *p* = 0.01); LNS scores with ITC of 40-Hz ASSR in the right temporal cortex (*r* = 0.13, *p* = 0.046) and left temporal cortex (*r* = 0.17, *p* = 0.02); WRAT scores with ITC of 40-Hz ASSR in the left temporal cortex (*r* = 0.17, *p* = 0.01); CVLT scores with ITC of 40-Hz ASSR in the left temporal cortex (*r* = 0.14, *p* = 0.04) and left superior frontal cortex (*r* = 0.17, *p* = 0.02)
**10**	Koshiyama et al. 2020c [67]	Healthy controls: 503; 234/269; 43.7 (12.8) Schizophrenia: 695; 477/218; 45.5 (10.2)	Letter-Number Sequencing (LNS), Letter-Number Span (LN Span), California verbal learning test (CVLT-2) list A 1–5 total score and Recognition Hits subscales, Reading subtest of the Wide Range Achievement Test-3 (WRAT)	40 Hz; clicks; 500 ms; 200 trains	EEG ITC and ERSP at Fz	40-Hz ITC and ERSP with LNS (*p* < 0.00042)
**11**	Lehongre et al. 2011 [68]	Healthy controls: 21; 11/10; 24.38 (3.85) Dyslexic: 23; 14/9; 24.61 (4.57)	Alouette test (reading fluency); Random Automatized Naming (RAN); composite measure of phonology (PHONO); the Wechsler Adult Intelligence Scale (WAIS-III), Digit span, Spoonerism tasks, Nonword repetition test	10–80 Hz; chirp; 5.4 s; 40 trials in 2 sessions, each with 80 sweeps	MEG Power, power asymmetry (left-right) at planum temporale (PT), superior temporal sulcus	Healthy controls: Reading speed and 30-Hz ASSRs power (*p* < 0.05) in left and right PT; Verbal fluency mean evoked power at 40-Hz *(r* = 0.223, *p* = 0.019). Composite measure of phonology and 30-Hz ASSRs power (*p* < 0.05) in left PT Dyslexic: Spoonerism task and 30-Hz ASSR power asymmetry (left minus right) (*r* = −0.450, *p* = 0.047) (effect was mostly driven by nonword repetition (*r* = 0.44, *p* = 0.04); RAN and 30-Hz ASSR power asymmetry (*r* = 0.552, *p* = 0.006); Digit span and 45–65 Hz magnitude in left PT (at 58-Hz *r* = −0.542, *p* = 0.009), left PFC (*r* = −0.486, *p* = 0.022) and left STS (*r* = −0.511, *p* = 0.015)
**12**	Leonhardt et al. 2019 [69]	Schizophrenia or schizoaffective disorder: 17; 14/3; 21.5 (3.8)	Brief Assessment of Cognition in Schizophrenia (BACS): Composite score	40 Hz; clicks; 500 ms; 80 trials	EEG Power; at Fz and Cz	Schizophrenia or schizoaffective disorder: n.s.
**13**	Light et al. 2006 [43]	Healthy controls: 80; n/a; 33.6 (9.95) Schizophrenia: 100; n/a; 42.5 (8.31)	The Wide Range Achievement Test 3 Reading subtest; California Verbal Learning Test (CVLT-2 list A, 1–5 total score and the delayed free recall indices); Wisconsin Card Sorting Test (WCST-64); Letter-Number Sequencing (LNS) test forward and reordering conditions	30, 40 Hz; clicks; 500 ms; 200 trials	EEG Evoked power and ITPC; at FCz	Healthy controls: n.s. Schizophrenia: LNS and power at 40-Hz (*r* = 0.32, *p* < 0.01)
**14**	Murphy et al. 2020 [70]	Healthy controls: 17; 9/8; 28.87 (5.98) Early-stage schizophrenia: 12; 12/0; 27.5 (6.89) Chronic-stage schizophrenia: 16; 13/3; 33.63 (6.94)	Brief Assessment of Cognition in Schizophrenia (BACS): Composite score	20 Hz, 30 Hz, 40 Hz; clicks; 1000 ms; 100 trails each of 10 block	MEG Amplitude and phase-amplitude coupling at combined region of bilateral transverse temporal cortex and superior temporal gyrus	Healthy controls: n.s. Early stage schizophrenia: n.s. Chronic stage schizophrenia: n.s.
**15**	Parčiauskaitė et al. 2019 [71]	Healthy subjects: 28; 28/0; 25.8 (3.3)	Psychology Experiment Building Language based task battery: Choice response time task (CRT), Stroop test (SOO), Tower of London test (TOL), Lexical decision task (LDT) and Semantic categorization task (SCT)	40 Hz; clicks; 500 ms; 150 trials each containing 20 clicks	EEG PLI and ERSP; the left (F3, F1, FC1, C1, FC3, C3), central (Fz, FCz, Cz), and right (F4, F2, FC2, C2, FC4, C4) regions	Healthy subjects: Tower of London task (number of moves) and 40-Hz PLI and ERSP: left (PLI: *r* = 0.55, *p* < 0.01; ERSP: *r* = 0.57, *p* < 0.01), center (PLI: *r* = 0.37, *p* = 0.05; ERSP: *r* = 0.42, *p* = 0.03) and right (PLI: *r* = 0.43, *p* = 0.02; ERSP: *r* = 0.46, *p* = 0.01) regions
**16**	Puvvada et al. 2018 [72]	Healthy controls: 108; 71/37; 37.9 (13.8) Schizophrenia: 128; 86/42; 37.8 (13.1) First-degree relatives of schizophrenia patients: 55; 17/38; 46.6 (13.6)	Digit sequencing task (digit span)	40, 80 Hz; clicks; 375 ms and 187.5 ms; 75 trials, each containing 15 clicks;	EEG Power and PLI; at frontocentral electrodes (AF3, AFZ, AF4, F3, F1, FZ, F2, F4, FC3, FC1, FCZ, FC2, FC4, C1, CZ, C2)	Healthy controls: n.s. Schizophrenia: Digit span and power at 40-Hz (*r* = 0.20, *p* = 0.033); First-degree relatives of schizophrenia patients: Digit span and power at 40-Hz (*r* = 0.42, *p* = 0.003)
**17**	Rass et al. 2010 [73]	Healthy controls: 77; 40/47; 41.0 (10.3) Euthymic bipolar disorder: 22; 43.6 (10.5) Acute bipolar disorder: 43; 42.6 (10.3)	Subtests from The Wechsler Abbreviated Scale of Intelligence (WAIS-III): Picture Completion, Digit Symbol Coding, Digit Span, Similarities	30, 40, 50 Hz; clicks; 467–480 ms; 80 trials	EEG MTP and PLF; at FCz	Healthy controls: n.s. Bipolar disorder: n.s.
**18**	Rass et al. 2012 [74]	Healthy controls: 56; 26/30; 38.75 (10.4) Schizophrenia and schizoaffective disorder: 42; 23/19; 36.86 (12.8) First-degree relatives of schizophrenia patients: 35; 13/22; 36.03 (12.5) Schizotypal personality disorder: 34; 20/14; 37.35 (9.2)	Subtests from The Wechsler Abbreviated Scale of Intelligence (WAIS-III): Picture Completion, Digit Symbol Coding, Digit Span, Similarities	30, 40, 50 Hz; clicks; 467–480 ms; 80 trials	EEG MTP and PLF; at FCz	Healthy controls: Similarities and 40-Hz PLF (*r* = 0.38, *p* < 0.01); Symbol Coding and 50-Hz MTP (*r* = 0.26, *p* = 0.03) Schizophrenia and schizoaffective disorder: Similarities and 40-Hz MTP (*r* = 0.34, *p* = 0.04), 40-Hz PLF (*r* = 0.34, *p* = 0.04); Digit span and 50-Hz PLF (*r* = 0.38, *p* = 0.02) First-degree relatives of schizophrenia patients: Similarities and 40-Hz PLF (*r* = 0.39, *p* = 0.03); Similarities and 50-Hz PLF (*r* = 0.45, *p* = 0.01) Schizotypal personality disorder: Similarities and 40-Hz MTP (*r* = 0.34, *p* = 0.04); Similarities and 50-Hz PLF (*r* = 0.40, *p* = 0.02)
**19**	Rojas et al. 2011 [75]	Healthy controls: 20; 7/13; 43.84 (6.86) Parents of children with ASD: 21; 6/15; 43.67 (7.33)	The Wechsler Abbreviated Scale of Intelligence (WAIS): Verbal IQ, Performance IQ, Full scale IQ	32, 40, 48 Hz; AM tone; 500 ms; 150 trials	MEG PLF, evoked, induced and total power: left and right hemispheres	Healthy controls: n.s. Parents of children with ASD: n.s.
**20**	Sun et al. 2018 [76]	Healthy controls: 30; 16/14; 34.2 (10.3) Schizophrenia: 24; 13/11; 33.0 (11.0)	MATRICS Consensus Cognitive Battery (MCCB) Chinese version: Trail Making Test: Part A, Symbol Coding Test, Animal Naming Test, Continuous Performance Test (Identical Pairs), Wechsler Memory Scale Third Edition (Spatial Span test and Letter-Number Span test), Hopkins Verbal Learning Test, Simple Visuospatial Memory Test, Mazes test, Mayer–Salovey–Caruso Emotional Intelligence Test (Managing Emotions)	40 Hz; clicks; 500 ms; 150 trains	EEG Power, PLF, ITPC; 128 electrodes	Healthy controls: Mazes test and PLF (*r* = 0.66); Mazes test and ITPC (*r* = 0.69); Trail Making Test: Part A and PLF (*r* = 0.56, *p* < 0.05); Trail Making Test: Part A and ERSP (*r* = 0.62, *p* < 0.05); Cognitive assessment total score and PLF (*r* = 0.48, *p* < 0.05); Cognitive assessment total score and ERSP (*r* = 0.59, *p* < 0.05) Schizophrenia: Mazes test and 40-Hz PLF (*r* = 0.55, *p* < 0.05); Mazes test and ITPC (*r* = 0.54, *p* < 0.05)
**21**	Tada et al. 2016 [44]	Healthy controls: 21; 11/10; 22.4 (3.3) First-episode schizophrenia: 13; 8/5; 24.5 (5.9) Ultra-high-risk individuals: 15; 9/6; 22.1 (4.0)	BACS-J: Verbal memory, Digit sequencing task (digit span), Token motor task, Category fluency, Letter fluency, Symbol coding, Tower of London	30, 40 Hz; clicks; 500 ms; 200 trials	EEG ITPC and ERSP; late latency at FCz	Healthy controls: n/a. First-episode schizophrenia: Symbol coding and the 40-Hz ITPC (*r* = 0.75, *p* = 0.003) and ERSP (*r* = 0.76, *p* = 0.003) Ultra-high-risk individuals: n.s.
**22**	van Deursen et al. 2011 [55]	Healthy controls: 20; 12/8; 69.5 (6.1) Mild Alzheimer’s disease: 15; 11/4; 75.2 (6.9) Mild cognitive impairment: 20; 12/8; 70.6 (7.2)	The cognitive subscale of the Alzheimer’s Disease Assessment Scale (ADAS-cog)	40 Hz; clicks; 450 ms; 80 trials	EEG Power; at T5, T6, O2, Fz, Pz, Cz	Combined sample: ADAS-cog and 40-Hz power at T5 (*r* = 0.43, *p* = 0.019) and T6 (*r* = 0.38, *p* = 0.028)

ERSP—event-related spectral perturbation, ITC—inter-trial coherence, ITPC—inter-trial phase coherence, MTP—mean trial power, PLI—phase-locking index, PLF—phase-locking factor, SNR—signal-to-noise ratio; n/a—not available; n.s.—not significant.

**Table 2:** The neurocognitive tasks/measures used in the included studies, grouped by domains.

	Domain	Assessment
1	Global cognition/ functioning or intellectual ability (*g*)	MCCB (Measurement and Treatment Research to Improve Cognition in Schizophrenia (MATRICS) Consensus Cognitive Battery), WAIS (Wechsler Adult Intelligence Scale), ADAS-cog (The Alzheimer’s Disease Assessment Scale–Cognitive Subscale), BACS (Brief Assessment of Cognition in Schizophrenia)
2	Attentional control and executive functions	Choice response task, Stroop test, Continuous Performance Test, Identical pairs, Serial addition task, Paced auditory serial addition task, Category fluency, Semantic categorization task, Semantic word list generation
3	Processing speed	Symbol search, Trial making test, Symbol coding, Symbol Digit Modalities Test, Rapid naming, Verbal fluency (letter, category), Picture completion
4	Short-term and working memory	List learning, Digit span, Spatial span, Letter–Number span, Letter–Number sequencing, Visuospatial Memory Test, Verbal memory, Hopkins Verbal Learning Test, Buschke Selective Reminding Test, Spatial recall test, California Verbal Learning Test (CVLT)
5	Cognitive flexibility and reasoning	Wisconsin Card Sorting Test, Similarities, Mazes test, Tower of London, Mayer–Salovey–Caruso Emotional Intelligence
6	Language abilities	Verbal IQ, Vocabulary, Reading, Non-word repetition, Auditory verbal learning, Spoonerism, Literacy, Random automatized naming, The Wide Range Achievement Test 3 Reading subtest, Lexical decision test, Alouette test
7	Motor abilities	Token task

## Data Availability

Not applicable.

## Supplementary Materials

The following are available online at https://www.mdpi.com/2076-3425/11/2/217/s1.
