# Supplementary material for: Gamma-Range Auditory Steady-State Responses and Cognitive Performance: A Systematic Review
Source: Brain Sci. 2021 Feb 10;11(2):217. doi: 10.3390/brainsci11020217 (PMC7916793; doi:10.3390/brainsci11020217)
Supplement: Supplementary file 1 [file brainsci-11-00217-s001.pdf]

Each study included in the Review (Table 1) was scored on individual items described in Table 3 to describe mainly recording bias. A study was scored 1 on an individual item when information was provided in full, 0.5 if the information was provided but some aspects were inconclusive or not mentioned, and 0 when the study did not provide the information on the item. Scores were further summed and a study with a total score of 5 and above was regarded as having a low risk of bias. Results of scoring are presented in Table 4.

Table 3. Description of reporting bias items**.**

	Bias	Description
**1**	Selection bias	Were cases and controls selected appropriately (e.g., appropriate diagnostic criteria or definitions, equal application of exclusion criteria to case and controls, sampling not influenced by exposure status)?
**2**	Performance bias	Did researchers rule out any impact from a concurrent intervention or an unintended exposure that might bias results?
**3**	Attrition bias	If attrition (overall or differential nonresponse, dropout, loss to follow-up, or exclusion of participants) was a concern, were missing data handled appropriately?
**4**	Detection bias	Were variables assessed/defined using valid and reliable measures, implemented consistently across all study participants?
**5**	Statistical power	Was sample size adequate?
**6**	Paradigm description	Was the description of the paradigm used provided in full, and replicable (type of paradigm, stimuli characteristics, presentation ratio, inter-stimulus interval, response type)?
**7**	Cognitive variables acknowledgement	Were correlations between cognitive evaluation and ASSR measures discussed and possible reasons attributed?

Table 4. Risk of reporting bias rating scores. Studies rated as high risk of bias (total score of 5 and less) are highlighted in grey. 0 score - high risk of bias (data on item not given, insufficient); 0.5 – medium risk bias (some data is missing); 1 - low risk of bias (all data is provided).

**Article**	**1**	**2**	**3**	**4**	**5**	**6**	**7**	**TOTAL**
Arrondo et al. 2009	1	1	1	1	1	1	1	7
Bartolomeo et al. 2019	1	1	1	1	0.5	0	0	4.5
Gaskins et al. 2019	0	0.5	1	0.5	0.5	0	1	3.5
Hirano et al. 2020	1	1	1	1	1	1	0	6
Hirtum et al. 2019	0.5	1	1	1	1	1	1	6.5
Kim et al. 2019	1	1	1	1	1	1	1	7
Kirihara et al. 2012	1	1	1	1	1	1	1	7
Koshiyama et al. 2020a	1	1	1	1	1	1	1	7
Koshiyama et al. 2020b	1	1	1	1	1	1	1	7
Koshiyama et al. 2020c	1	1	1	1	1	1	1	7
Lehongre et al. 2011	0.5	0.5	1	1	1	1	1	6
Leonhardt et al. 2019	1	1	1	1	0.5	1	1	6.5
Light et al. 2006	0.5	1	1	1	1	1	1	6.5
Murphy et al. 2020	1	1	1	1	1	1	0.5	6.5
Parčiauskaitė et al. 2019	1	1	1	1	1	1	1	7
Puvvada et al. 2018	1	1	1	1	1	1	1	7
Rass et al. 2010	1	1	1	1	1	1	0.5	6.5
Rass et al. 2012	1	1	1	1	1	1	0	6
Rojas et al. 2011	1	0	1	1	1	1	1	6
Sun et al. 2018	1	1	0	1	1	1	1	6
Tada et al. 2016	1	1	1	0.5	0.5	1	1	6
van Deursen et al. 2011 [39]	1	1	1	1	0.5	1	1	6.5
